# Novel Systemic Therapies in Advanced Liposarcoma: A Review of Recent Clinical Trial Results

**DOI:** 10.3390/cancers5020529

**Published:** 2013-05-10

**Authors:** William W. Tseng, Neeta Somaiah, Alexander J. Lazar, Dina C. Lev, Raphael E. Pollock

**Affiliations:** 1 Department of Surgical Oncology, The University of Texas M.D. Anderson Cancer Center, 1515 Holcombe Blvd, Houston, TX 77030, USA; E-Mail: wtseng@mdanderson.org; 2 Department of Sarcoma Medical Oncology, The University of Texas M.D. Anderson Cancer Center, 1515 Holcombe Blvd, Houston, TX 77030, USA; E-Mail: nsomaiah@mdanderson.org; 3 Department of Pathology, The University of Texas M.D. Anderson Cancer Center, 1515 Holcombe Blvd, Houston, TX 77030, USA; E-Mail: alazar@mdanderson.org; 4 Department of Cancer Biology, The University of Texas M.D. Anderson Cancer Center, 1515 Holcombe Blvd, Houston, TX 77030, USA; E-Mail: dlev@mdanderson.org

**Keywords:** liposarcoma, chemotherapy, molecular-based therapy, clinical trials

## Abstract

Liposarcoma is one of the most common adult soft tissue sarcomas an consists of three histologic subtypes (well and dedifferentiated, myxoid/round cell, and pleomorphic). Surgery is the mainstay of treatment for localized disease; however for unresectable or metastatic disease, effective treatment options are currently limited. In the past decade, a better understanding of the distinct genetic and molecular aberrations for each of the three histologic subtypes has led to the development of several novel systemic therapies. Data from phase I and early phase II clinical trials have been reported. Despite challenges with conducting clinical trials in liposarcoma, preliminary results for several of these novel, biology-driven therapies are encouraging.

## 1. Introduction

Soft tissue sarcomas are a heterogeneous group of over 50 different malignancies of mesenchymal origin [1]. In adults, liposarcoma is one of the most common types of soft tissue sarcoma [2]. Within liposarcoma, three distinct histologic subtypes are recognized by the World Health Organization: (1) well and dedifferentiated, (2) myxoid/round cell, and (3) pleomorphic liposarcoma [2]. Although surgical resection is the mainstay of treatment for localized disease in all subtypes, many patients with liposarcoma will initially present with or ultimately progress to advanced disease that is either unresectable, metastatic or both. For these patients, the mortality is high and local and/or systemic tumor burden may also cause significant morbidity. As a method of local control, radiation therapy can provide symptom palliation for a small subset of patients, but systemic disease is not addressed [3,4]. Current cytotoxic chemotherapy can potentially provide systemic control, but toxicity tends to be quite high [3,4]. As a result, for the majority of liposarcoma patients with advanced disease, treatment options are currently limited. 

In this review, we will provide an overview of liposarcoma followed by a brief discussion of conventional cytotoxic chemotherapy and commonly adopted endpoints for treatment efficacy. We will then summarize the results of recent clinical trials with novel systemic therapies for patients with advanced liposarcoma. Many of published results are from studies done in the context of the broader group of adult soft tissue sarcoma; however we will focus on liposarcoma. As we will discuss, even within liposarcoma, recognition of the specific histologic subtype is critical, especially as novel therapies are emerging based on a better understanding of subtype-specific disease biology.

## 2. Three Distinct Liposarcoma Subtypes

In contrast to benign lipomas, all three subtypes of liposarcoma are true adipocytic malignancies which can cause significant morbidity and mortality. Each subtype is characterized by distinct genetic and molecular aberrations and unique histologic appearance, suggesting separate pathways to malignant transformation [2,3,4]. Accordingly, the initial presentation, pattern of disease progression and overall clinical outcome varies with each liposarcoma subtype. An understanding of these multi-level differences (Table 1) is critical to the management of the liposarcoma patient and selection of appropriate treatment options.

**Figure 1:**
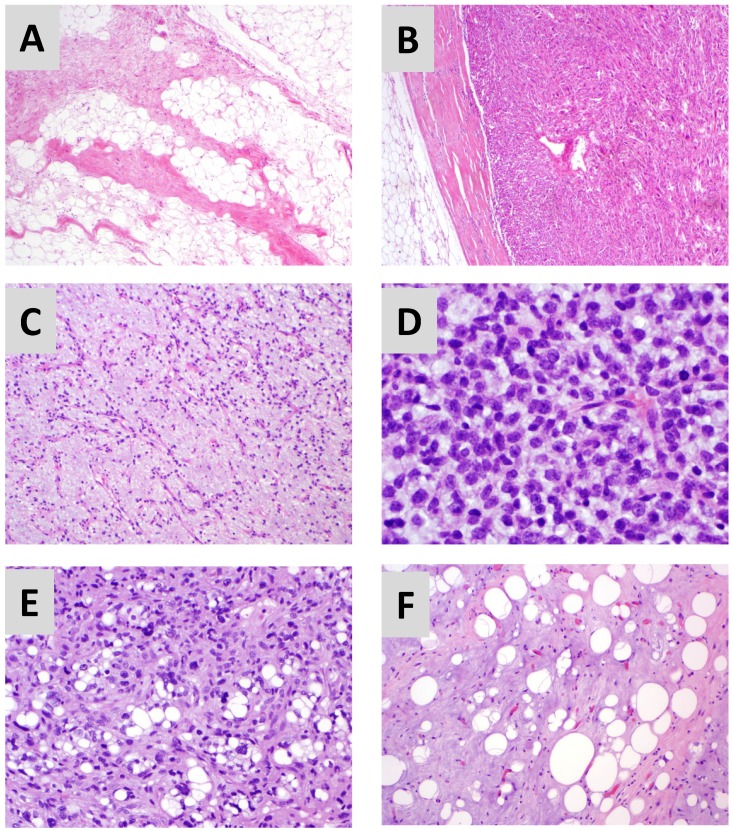
Representative photomicrographs of well differentiated (**A**), dedifferentiated (**B**), myxoid (**C**)/round cell (**D**), and pleomorphic (**E**) liposarcoma. A challenging case is shown in (**F**). This portion of a well differentiated liposarcoma shows myxoid features, however other areas of the tumor showed characteristic features and 12q13-15 amplification.

Well-differentiated (WD) and dedifferentiated (DD) liposarcoma are the most common subtype of liposarcoma [2]. Both WD and DD tumor cells exhibit amplification of chromosome 12q13-15, a region which contains several hundred genes including MDM2, an inhibitor of the tumor suppressor p53, and CDK4, a critical regulator of cell cycling. By histology, WD liposarcoma are characterized by the presence of adipocytes oma (Figure 1A); lipoma-like, sclerosing, and inflammatory variants have also been described [2]. DD liposarcomas typically have an adipocyte-rich, WD portion that is well demarcated from a highly cellular, spindle cell-rich DD portion (Figure 1B). To establish a definitive histologic diagnosis of DD, five or more mitoses per 10 high power fields are required [2]. The majority of DD cases are found *de novo*, but up to 25–40% of patients with WD will ultimately manifest DD histology at recurrence [5]. DD liposarcoma was traditionally thought to arise from WD; however the exact clonal relationship between WD and DD liposarcoma is not clear. Presence of DD histology nonetheless is associated with much more aggressive disease and worse clinical outcome [5,6].

**Table 1:** Important multi-level differences in liposarcoma subtypes.

Liposarcoma Histologic Subtype	Genetic and/or Molecular Aberration	Histologic Features	Anatomic Site	Clinical Behavior	Response to Current Therapy
**Well differentiated (WD)**	12q13-15 amplification (MDM2, CDK4, *etc*.)	Adipocytes of varying size, prominent fibrous stroma	Retroperitoneum >extremities, paratesticular areas, trunk	Locoregional recurrence	Poor
**Dedifferentiated (DD)**	same as WD	Highly-cellular portion (5 or more mitoses/10 HPF) next to WD portion	same as WD	Locoregional recurrence and distant metastasis (10–15%)	Low
**Myxoid/Round Cell (MRC)**	Translocation (t12;16)(q13;p11) or (t12;22)(q13;q12) leading to FUS-CHOP/DDIT3 or EWS-CHOP/DDITS fusion protein	Abundant extracellular myxoid material; sparse cellular portion w/mature adipocytes, immature lipoblasts, round cells (>5% of tumor)	Proximal lower extremities	Distant metastasis (10–20%) to visceral organ sites, bone, and fat bearing areas	High
**Pleomorphic**	Complex	Highly cellular resembling MFH; pleomorphic lipoblasts; occassional multinucleated cells	Lower extremities >retroperitoneum; mediastinum	Distant metastasis (30–50%)	Low

WD and DD liposarcoma does not have a certain age or gender predilection and there are no known risk factors (e.g., obesity) for the development of disease. WD and DD liposarcoma most commonly occur in the retroperitoneum and to a lesser extent, the extremities, paratesticular areas, and trunk. WD liposarcoma does not metastasize, whereas DD liposarcoma has the potential for distant metastasis, typically to the lungs. The true frequency of this event, however, has been estimated to be 10–15% [7]. For both WD and DD, the majority of patients experience locoregional morbidity. In the retroperitoneum, tumors can be massive in size (>30 cm) and/or invade adjacent viscera and structures, making surgical resection challenging. Locoregional recurrence is very common and patients often undergo multiple re-operations. WD and DD liposarcoma are largely resistant to conventional cytotoxic chemotherapy and radiation therapy [2,3,4], and as a result, treatment options other than surgery, are limited. 

Myxoid/round cell (MRC) liposarcoma is the second most common subtype of liposarcoma. In terms of genetic abnormalities, MRC liposarcoma is characterized by translocation of chromosomes 12 and 16 (t12;16)(q13;p11), that results in a fusion gene arrangement between FUS and CHOP/DDIT3. In rare cases, an alternative translocation event can occur (t12;22)(q13;q12), that results in an EWS-CHOP fusion gene. FUS- and EWS-CHOP/DDIT3 are both thought to interfere with normal adipocytic differentiation through the C/EBP family of transcription factors and are likely involved in activation of a number of tyrosine kinase receptor pathways including MET, RET and PI3K/Akt. By histology, MRC liposarcoma is notable for abundant extracellular myxoid material with typically, a sparse cellular component consisting of mature adipocytes, immature lipoblasts and round cells (Figure 1C) [2]. In a subset of cases, the cellularity increases with a predominance of round cells containing a high nuclear to cytoplasmic ratio (Figure 1D). Round cell transformation—defined as >5% of the tumor—is associated with more aggressive disease biology and worse clinical outcome [8].

MRC liposarcoma tends to present in younger patients and affects the proximal lower extremities as opposed to the retroperitoneum. In fact, intraabdominal and retroperitoneal disease may actually be WD/DD liposarcoma that was misdiagnosed [9]. Distant dissemination to visceral organ sites (e.g., lung) occurs in 10–20% of patients, especially with round cell transformation. Patients should be carefully monitored for skeletal metastases (e.g., w/MRI), which may represent up to half of all metastatic events [10]. Unique among the liposarcoma subtypes, MRC liposarcoma also appears to have a predilection for metastases to fat-bearing areas in the retroperitoneum, chest, trunk and other extremities [11,12]. Several studies have established that tumors at distant fat-bearing sites are in fact, metastases from the same clonal origin as the primary site, as opposed to multifocal sites of disease [13,14]. Treatment for MRC liposarcoma consists of surgical resection for localized, primary disease. In patients with advanced or metastatic disease, MRC liposarcoma is known for its sensitivity to radiation therapy [11] and cytotoxic chemotherapy [15] in comparison to the other liposarcoma subtypes [16].

Pleomorphic liposarcoma is the third, least common and least understood subtype of liposarcoma. A single characteristic genetic abnormality has not yet been identified; instead, complex changes are seen with chromosomal duplications, gains, losses, and rearrangements. By histology, pleomorphic liposarcoma resembles a non-adipocytic soft tissue sarcoma called malignant fibrous histiocytoma (MFH), also known as undifferentiated pleomorphic sarcoma (UPS), with high cellularity and additionally, presence of pleomorphic lipoblasts and occasional multinucleated giant cells (Figure 1E) [2]. An epithelioid histologic variant resembling renal cell or adrenocortical carcinoma has also been described [17]. Patients with pleomorphic liposarcoma most commonly present with disease in the lower extremity and occasionally at other sites, including the retroperitoneum and mediastinum. Disease progression is much more aggressive compared to the other liposarcoma subtypes, with a higher (30–50%) frequency of distant metastasis to visceral organ sites, including lung, bone and liver. Tumors are highly resistant to all current treatment modalities [18,19].

Despite hallmark genetic aberrations and characteristic histologic features, in practice, distinguishing one liposarcoma subtype from another can at times be challenging (Figure 1F). Histologic examination, ideally by an experienced soft tissue sarcoma pathologist, is best supplemented with molecular studies (e.g., MDM2 amplification by fluorescence in situ hybridization in WD/DD liposarcoma) for accurate diagnosis. Molecular studies can frequently lead to reclassification of incorrectly diagnosed cases [20,21]. An accurate diagnosis is critical not only to counsel patients regarding likely disease course (e.g., frequent locoregional recurrence in retroperitoneal WD/DD liposarcoma; metastases to fat-bearing areas in MRC) but for decision making with regard to available treatment options or enrollment into clinical trials for those with advanced disease.

## 3. Conventional Cytotoxic Chemotherapy

The current cytotoxic chemotherapy agents for unresectable/metastatic liposarcoma are based on efficacy data from trials encompassing all soft tissue sarcoma subtypes. Single agent anthracycline (mainly doxorubicin) or an anthracycline-based combination is considered the standard for first-line therapy in patients with advanced disease [22]. Other agents with single agent activity that are frequently combined with doxorubicin are ifosfamide and dacarbazine. The objective response rate (ORR) in treatment naïve soft tissue sarcoma patients is somewhere between 18 to 38% [23,24,25,26,27,28,29,30]. Although anthracycline based combinations tend to have higher response rates compared to single agent therapy, a survival benefit has not been demonstrated in the trials so far [25,26]. This might be due to the studies having inadequate power to detect the survival advantage or due to the higher toxicity and limited added efficacy of the cytotoxic agent used in the combination. 

Gemcitabine and docetaxel is a frequently used non-anthracycline combination in the second-line setting for liposarcomas. A randomized phase II study conducted by Maki *et al.* in metastatic soft tissue sarcoma suggested a survival benefit for fixed-dose rate gemcitabine with docetaxel over fixed-dose rate gemcitabine alone [31]. The median progression-free survival (PFS) and overall survival (OS) of gemcitabine therapy alone was 3 months and 18 months compared to 6 months and 12 months with the combination, respectively. The best responses were seen in leiomyosarcoma and undifferentiated pleomorphic sarcoma (UPS/MFH) patients. Among the small number of liposarcoma patients (n = 20, 16% of all patients), only two (both WD/DD) had stable disease at 6 months with gemcitabine alone. An additional five patients (three WD/DD, two MRC) with gemcitabine alone and five patients (four WD/DD, one pleomorphic) with combination therapy had stable disease for less than 6 months. 

To date, no prospective trial with conventional cytotoxic chemotherapy agents has individually assessed response in a liposarcoma patient cohort alone. Retrospective, subtype specific studies have been reported which reflect response in liposarcoma patients and also highlight the variability between the three major liposarcoma subtypes. In these studies, the combination of doxorubicin and ifosfamide resulted in good response rates in MRC liposarcoma (ORR = 43%) and is hence the treatment of choice for this subtype [32]. Italiano *et al*. reported a multicenter, retrospective study of 208 WD and DD liposarcoma patients, 82% of which were treated with an anthracycline-containing regimen. The ORR was only 12% and all of the responses occurred in anthracycline treated patients. Rates of 3- and 6 month PFS were 59% and 44% [33]. In pleomorphic liposarcoma, Italiano *et al*. reported an ORR of 37% with various cytotoxic chemotherapy regimens with no significant difference between the various single agents or combination regimens used [34]. Of note, only 32 patients over a ten year period were assessable for response, attesting to the rarity of this disease. Rates of 3- and 6 month PFS were 63% and 43%.

For all soft tissue sarcomas in general, despite objective response rates, a median survival of 8 to 13 months is estimated from the start of first-line anthracycline-based chemotherapy, as shown in randomized studies performed over the last two decades [24,27,30,35]. The median survival for patients for whom conventional chemotherapy with an anthracycline and ifosfamide has failed is in the range of 6 months [28,36,37]. Most recent studies therefore, focus on the progression free rate (PFR) when evaluating agents for anti-tumor activity. A commonly used reference benchmark for an active agent in soft tissue sarcoma was proposed by Van Glabbeke *et al*. [38]. Based on analysis of fourteen clinical trials of cytotoxic therapies conducted by the EORTC, the PFR for active and inactive agents for soft tissue sarcomas (all histologies together) in the first and second line setting was determined. For first-line therapy, a 6-month PFR of 30% or higher suggested drug activity. For second-line therapy, a 3-month PFR of 40% or higher was associated with an active drug and 20% or lower meant inactivity. A recent review of published series confirmed that ifosfamide, dacarbazine-gemcitabine and docetaxel-gemcitabine meet these disease stabilization criteria as second-line therapies [22].

## 4. Novel Systemic Therapies in Liposarcoma

In the past decade, results from clinical trials have identified several novel systemic therapies in soft tissue sarcoma, many of which have potential efficacy in liposarcoma (Table 2). In contrast to conventional cytotoxic chemotherapies, which are non-specific, the majority of these novel therapies are based on the understanding of disease biology inherent to a given sarcoma histology, in many cases targeting a specific, aberrant genetic or molecular pathway. 

**Table 2:** Overview of reported human studies and clinical trials for novel systemic therapies in liposarcoma.

Novel Therapy	Mechanism of Action	Liposarcoma Histologic Subtype	Study Type/Clinical Trial Phase	References^ (n = liposarcoma pts)
**Trabectedin**	Binding of DNA minor groove; direct interaction w/FUS-CHOP	MRC	Phase II, Retrospective, and Neoadjuvant	Garcia-Cabonero, 2004 (10); Yovine, 2004 (6); Le Cesne, 2005 (10); Grosso, 2007 (51 *); Grosso, 2009 (32 *); Demetri, 2009 (93); Gronchi, 2012 (23 *), Samuels, 2013 (233)
**Eribulin**	Microtubule inhibitor	DD	Phase II	Schoffski, 2011 (37)
**RG7112**	MDM2 antagonist	WD/DD	Phase I (Neoadjuvant)	Ray-Coquard, 2012 (20 *)
**Flavopiridol**	pan-CDK inhibitor, including CDK4	WD/DD	Phase I	Luke, 2012 (16)
**PD 0332991**	CDK4/6 inhibitor	WD/DD	Phase I	Schwartz, 2012 (7)
**Troglitazone, Rosiglitazone, Efatutazone**	PPAR-gamma agonist	all	Phase I, II	Debrock, 2003 (12 *); Pishvaian, 2012 (5)
**Nelfinavir**	SREBP-1 inhibitor	WD/DD	Phase I	Pan, 2012 (20 *)
**Pazopanib, Sorafenib, Sunitinib**	Tyrosine kinase receptor inhibitor	all	Phase II	Sleijfer, 2009 (19); von Mehren, 2012 (10); Tariq Mahmood, 2011 (17)

^ = reports with n = 5 or less patients were excluded, but are described in the text. * = indicates the study was focused on liposarcoma only.

### 4.1. Marine-Derived Compounds—Trabectedin and Eribulin

Trabectedin (Yondelis^TM^/Ecteinascidin-743) is a tetrahydroisoquinoline alkaloid compound, originally derived from the Carribean sea tunicate, *Ecteinascidia turbinata* [39]*.* Among the novel systemic therapies in liposarcoma, trabectedin is the most well-studied in clinical trials and thus far, has the highest treatment efficacy. 

The primary mechanism of action for trabectedin is through binding of the DNA minor groove, causing structural changes and irreversible DNA damage that leads to cell cycle arrest and apoptosis. Mechanistic studies have demonstrated that trabectedin may have target specificity in MRC liposarcoma by direct interaction with the FUS-CHOP fusion protein, preventing its binding to transcriptional promoters and restoring normal lipoblast maturation [40]. Recent studies also suggest that trabectedin may have anti-inflammatory effects and specifically target tumor-associated macrophages [41,42].

Three prospective phase II studies initially established the therapeutic potential of trabectedin in patients with advanced soft tissue sarcomas. A closer scrutiny of enrolled patients in these studies, however, demonstrates that very few actually had liposarcoma. Garcia-Carbonero *et al*. reported on 36 patients, only 10 (28%) of which had liposarcoma (histologic subtypes not specified) [43]. Nonetheless, major objective responses were seen in two of these patients (one MRC, one DD), including complete response in the patient with MRC liposarcoma. Yovine *et al*. had only six liposarcoma patients (11% of total) in their study, one of whom had stable disease for 31 months [44]. Le Cesne *et al*. had 10 liposarcoma patients (10% of total), four of whom had either stable disease or partial response [45]. 

Demetri *et al*. subsequently reported results of a larger phase II study which compared two different dosing schedules of trabectedin (intravenous administration over 3 h every week *vs*. 24 h every 3 weeks) in patients with leiomyosarcoma or liposarcoma, the latter of which had 93 patients (34%, subtype not specified) [46]. Trabectedin given for 24 h every 3 weeks was found to be superior, with 3 month PFR of 52% and 6 month PFR of 36% for all study patients; subanalysis of the liposarcoma patients alone was not reported.

Recently, Samuels *et al*. reported on RECIST objective response rates and overall survival for trabectedin in previously-treated patients with advanced soft tissue sarcoma, including 233 patients with liposarcoma (21% of total, subtype not specified), representing the largest series to date [47]. ORR for liposarcoma patients in this study was 6%, including seven patients with partial response and one patient with complete response. Patients with liposarcoma had a median overall survival of 18.1 months, compared to 11.9 for all evaluable patients. PFR was not reported.

The treatment efficacy of trabectedin was evaluated specifically for MRC liposarcoma in a multicenter, retrospective study reported by Grosso *et al.* [48]. Data was analyzed from 51 previously-treated patients with locally-advanced and metastatic disease who were enrolled in a compassionate use program. Remarkably, the PFR at 3 months was 92% and at 6 months, 88%. In addition, by RECIST criteria, 24 patients experienced partial response and two patients had complete response (ORR = 51%). The authors noted that in the majority of responders, changes in tissue density were seen on radiographic imaging prior to tumor shrinkage. Subsequent long-term analysis in the subset of 32 patients from the Milano group confirmed the durability of these results [49].

Use of trabectedin in the neoadjuvant setting for MRC liposarcoma was reported by Gronchi *et al*. [50]. In this multicenter phase II trial, 23 previously untreated patients received trabectedin prior to surgical resection. No disease progression was seen in any of the patients prior to surgery and 24% of patients experienced a RECIST objective response. Patients received a minimum of 3 and maximum of 6 cycles, each lasting 3 weeks, prior to a 3 week washout period after the last cycle leading up to surgery. Therefore, disease was monitored for a minimum of 3 months (3 × 3 + 3 = 12 weeks) in all study patients. Pathologic complete response was observed in 13% of patients as defined by complete absence of FUS-CHOP/DDIT3 translocation-positive tumor cells in the resection specimens. 

At the now standard dose of 1.5 mg/m^2^ given as a 24 h continuous infusion every 3 weeks, established by Demetri *et al*. [46], trabectedin is overall fairly well tolerated [51]. Fatigue and nausea are the most common subjective complaints. Biochemical transaminitis without clinical manifestations is frequently seen and transient neutropenia is the major dose limiting toxicity, but occurs in only a minority of patients. Rare cases of fulminant hepatic failure and rhabdomyolysis have been reported. 

Trabectedin was approved by the European Union in 2007 as second-line therapy for use in patients with soft tissue sarcoma and disease progression despite previous doxorubicin and ifosfamide treatment. In the United States, two phase III studies with trabectedin for advanced MRC liposarcoma are currently in progress (NCT01692678, NCT01343277). 

Interestingly, another marine-derived compound, eribulin mesylate, was recently reported to also have selective activity in liposarcoma. In a phase II study of four soft tissue sarcoma types, 37 patients (29% of total) with adipocytic sarcoma treated with eribulin demonstrated a 3 month PFR of 47%, the highest among the four cohorts [52]. PFR at 6 months was not reported. The majority (65%) of the patients in the adipocytic sarcoma cohort actually had DD liposarcoma. Eribulin is a microtubule inhibitor but to our knowledge, a liposarcoma specific mechanism of action has not been described.

### 4.2. MDM2 Antagonists

In well differentiated (WD) and dedifferentiated (DD) liposarcomas, amplification of MDM2 is seen in virtually all tumors and in fact, is a reliable method for clinical diagnosis [2]. This observation and the knowledge of the important role of MDM2 as a negative regulator of p53, suggest that targeting MDM2 may be a promising approach to therapy, specifically for WD/DD liposarcoma. Preclinical studies using Nutlin-3A, a selective MDM2 antagonist, restored p53 activity in liposarcoma cells leading to preferential induction of cell cycle arrest and apoptosis [53,54]. 

Ray-Coquard *et al*. recently reported the first clinical trial of an MDM2 antagonist specifically in patients with WD and DD liposarcoma [55]. Twenty previously untreated patients from four centers in France were enrolled in a phase I, proof-of-mechanism study of the oral MDM2 antagonist, RG7112, given in the neoadjuvant setting. Analysis of resected tumors in these patients demonstrated restoration of p53 and downstream p21 expression as well as statistically significant reduction in Ki67-positive, proliferating tumor cells. A correlation was also noted between MDM2 antagonist treatment and increased numbers of TUNEL-positive, apoptotic tumor cells; however this failed to reach statistical significance. Six patients (30%) experienced Grade 4 out of five neutropenia and three patients (15%) had thrombocytopenia; almost all study patients experienced nausea, vomiting and fatigue. During the limited duration of neoadjuvant therapy (up to three 28-day cycles), the majority of patients (70%) had stable disease and one patient had a partial response by RECIST criteria.

### 4.3. CDK4 Antagonists

Similar to MDM2, cyclin dependent kinase-4 or CDK4 is also consistently amplified in WD and DD liposarcoma and represents another appealing target for therapy for this histologic subtype. Mechanistically, CDK4 phosphorylates and functionally inactivates the retinoblastoma (Rb) protein, which results in uninhibited cell cycle progression from G1 to S phase. CDK4 inhibition would thus restore native cell cycle regulation and prevent uncontrolled tumor cell proliferation. 

Inhibitors of the broad family of CDKs have been tested for therapeutic value in both hematologic and solid tumors, with some compelling data for treatment efficacy in germ cell tumors and chronic lymphoid leukemia. One observation from these studies was that CDK-inhibitors may actually be effective as potentiators of cytotoxic chemotherapy agents. Luke *et al*. at the Memorial Sloan Kettering Cancer Center confirmed this concept in mouse xenograft models of soft tissue sarcoma, including DD liposarcoma, combining doxorubicin with the pan-CDK inhibitor, flavopiridol [56]. The authors then enrolled 31 patients with advanced soft tissue sarcoma in a phase I dose-escalation study of flavopiridol in combination with fixed dose doxorubicin. Fifteen patients (48% of total) had WD or DD liposarcoma and one patient had pleomorphic liposarcoma. Overall, toxicities were mostly hematologic (neutropenia, thrombocytopenia) and low to moderate grade; no maximum tolerated dose was reached. WD/DD liposarcoma patients demonstrated reasonable treatment efficacy. Among the 12 evaluable patients in this cohort, seven had stable disease at 12 weeks (PFR @ 3 mo = 58%) and 3 at 24 weeks (PFR @ 6 mo = 25%). One patient had stable disease for 99 weeks. 

The first in-human study of PD 0332991, an oral CDK4/6 specific inhibitor, was reported by Schwartz *et al.* [57]. Patients were enrolled in this phase I study if they had either non-Hodgkin’s lymphoma or Rb-positive advanced solid tumors including WD/DD liposarcoma. Dose-limiting hematologic toxicities were observed in six patients (18%) for the entire cohort. Stable disease was observed in four out of seven patients with liposarcoma including one patient with a durable, toxicity-free response (>23 cycles), despite previous progression on a tyrosine kinase receptor inhibitor. The authors have now opened a phase II study of PD 0332991, specifically for patients with Rb-positive liposarcoma (NCT01209598).

### 4.4. PPAR-Gamma Agonists

Peroxisome proliferator-activated receptors (PPAR) are critical regulators of normal adipocyte differentiation. PPAR-gamma is one of three isoforms that forms a heterodimeric complex with the retinoid X receptor to regulate transcription of adipocyte-specific genes involved in the terminal adipocyte differentiation pathway. In human liposarcoma cells, PPAR-gamma agonist not only induced adipocyte differentiation but demonstrated anti-tumor activity *in vitro* [58,59]. Activation of PPAR-gamma thus represents an attractive target particularly for DD, MRC and pleomorphic liposarcoma, as a mechanism to revert these subtypes to a more well differentiated phenotype with potentially more indolent disease progression, and for its direct anti-tumor activity.

Despite a plausible biologic basis, reported human studies using PPAR-gamma agonists for liposarcoma have thus far had mixed results with low numbers of enrolled patients. Demetri *et al*. reported a proof-of-mechanism study conducted at the Dana Farber Cancer Institute in three liposarcoma patients (2 MRC, 1 pleomorphic), using the anti-diabetic thiazolidinedione drug, troglitazone [59]. Tumor biopsies and mass-spectroscopy imaging demonstrated histologic and biochemical differentiation with accumulation of lipid droplets. The authors reported favorable safety and tolerability, however no data on anti-tumor activity was provided. Debrock *et al*. subsequently reported results from a phase II trial of rosiglitazone in 12 patients with DD and MRC liposarcoma [60]. Histologic and biochemical proof-of-mechanism was negligible and no clinical response was seen with a mean time to disease progression of 6 months. More recently, Pishvaian *et al*. reported more encouraging results with a phase I study of efatutazone, a newer generation PPAR-gamma agonist [61]. Five out 31 patients (16%) enrolled had liposarcoma (subtypes not specified) and one patient with MRC had a durable partial response for 690 days while on therapy. *In vitro* studies suggest that inhibition (as opposed to activation) of the PPAR-delta isoform may have anti-proliferative effect specifically for liposarcoma [62].

### 4.5. Nelfinavir

Use of HIV protease inhibitors (PI) has been linked to a clinical syndrome of lipodystrophy in which treated patients demonstrate peripheral fat atrophy and central fat accumulation, along with insulin resistance and hyperlipidemia. Alterations in sterol regulatory element binding protein-1 (SREBP-1), a master transcriptional regulator of fatty acid and cholesterol synthesis, are thought to be the underlying mechanism for HIV PI lipodystrophy. Liposarcoma cells were shown to express SREBP-1 and investigators at the City of Hope Medical Center performed *in vitro* studies to show selective, dose-dependent anti-proliferative and pro-apoptotic activity of HIV PIs [63,64]. Among several HIV PIs tested, the most potent effects were seen with nelfinavir, which was subsequently chosen for clinical trial testing. Pan *et al*. recently reported results of a phase I trial of nelfinavir conducted in 20 patients with unresectable liposarcoma, 17 of whom had WD/DD, two MRC and one pleomorphic subtypes [65]. Although one patient had grade 3 pancreatitis, no other dose-limiting toxicities were seen. One patient with DD experienced a partial response for 14 months and four additional patients had stable disease. A phase II trial of nelfinavir in advanced liposarcoma was also conducted (NCT00233948) but the results have yet to be reported.

### 4.6. Tyrosine Kinase Receptor Inhibitors

Tyrosine kinase receptors (TKRs) are a diverse family of surface molecules recognized for their critical role in regulating multiple aspects of carcinogenesis, tumor cell proliferation and disease progression (e.g., angiogenesis, metastasis) across many solid tumor types. In the presence of a specific growth factor ligand, TKRs dimerize and initiate downstream, intracellular signaling pathways. In tumor cells, TKRs and their associated downstream molecules are frequently over-expressed or mutated, leading to constitutive activation or aberrant signaling. 

Sleijfer *et al*. reported a large phase II study in patients with advanced soft tissue sarcoma, looking at pazopanib, an oral multi-targeted tyrosine kinase inhibitor with activity against VEGF, PDGF and KIT [66]. Sarcoma histology was diverse and 142 patients were included in the study, stratified by histology: liposarcoma, leiomyosarcoma, synovial sarcoma, and “other”; however, enrollment for patient with liposarcoma was stopped early because of lack of anti-tumor activity for this particular histology. Final analysis of 19 liposarcoma patients (subtypes not specified) demonstrated 3 month PFS in five out 19 of these patients (PFR = 26%). To determine if any liposarcoma subtypes have any clinical benefit with pazopanib, another phase II study specific for advanced liposarcoma patients is currently open (NCT01506596).

The Southwestern Oncology Group conducted a phase II study of sorafenib, a similar multitargeted anti-angiogenic TKR inhibitor in patients with vascular sarcomas, liposarcoma (10 patients = eight DD and two MRC) and leiomyosarcoma [67]. Von Mehren *et al*. reported that no tumor responses were seen in any of the patients. Consistent with the pazopanib trial data, patients with liposarcoma had the worst results with 3 month PFR of 30%. Maki *et al*. reported a larger phase II trial of sorafenib in 145 patients with soft tissue sarcomas; however there were only three patients with liposarcoma, placed into the “other” category for analysis [68].

Sunitinib is another TKR that has similar targets as pazopanib and sorafenib and is also regarded as an anti-angiogenic agent. Interestingly, in a single institution study of 48 patients at the Moffitt Cancer Center, Tariq Mahmood *et al*. reported impressive efficacy with sunitinib specifically for liposarcoma [69]. Out of 17 evaluable patients with liposarcoma (subtypes not specified), 14 (=82%) had stable disease). Three month PFR was 75% for untreated and 63% for previously treated liposarcoma patients, better results compared to the other histologies studied, leiomyosarcoma and UPS/MFH. Six month PFR was not reported. Sunitinib was well tolerated with Grade 1 or 2 hematologic and non-hematologic toxicities in the majority of patients. George *et al*. also reported a phase II trial of sunitinib in 53 patients with non-gastrointestinal stromal tumor sarcomas [70]. Only two patients had liposarcoma; one of whom had stable disease for 28 weeks.

It is unclear why sunitinib, but not pazopanib or sorafenib, has anti-tumor activity in liposarcoma despite similar molecular targets. The specific histologic subtype of liposarcoma patients enrolled (e.g., more indolent, WD liposarcoma), often not specified in the trials discussed, would have affected the interpretation of apparent drug efficacy. There may also be differential sensitivity to TKRs that is subtype-specific. Alternatively, in comparison to pazopanib or sorafenib, sunitinib may have selective affinity for other less well characterized TKRs targets that play an important role in tumor progression in liposarcoma. Recent work from our own group using an experimental *in vitro* and xenograft models of WD/DD liposarcoma has identified over-expression of several TKRs including EGFR, MET, AXL and IGFR, all of which may be potential targets [71]. 

### 4.7. Immunotherapy

Immunotherapy has recently gained much attention from success in large phase III trials with patients with metastatic melanoma, in which durable improvement in overall survival can be seen in patients that respond to treatment [72,73]. The immune system is capable of selectively recognizing and killing tumor cells with the theoretical advantage of minimal collateral damage to normal, non-malignant cells resulting in low toxicity for the patient [74]. Mechanistically, this is accomplished by natural or induced priming (e.g*.*, vaccination) of cytotoxic CD8 T cells against surface antigens that are selectively expressed on tumor cells. Our group has reported preliminary studies to suggest that a natural immune response exists in a subset of WD liposarcoma that may be amenable to immunotherapeutic strategies used in metastatic melanoma [75]. 

In MRC liposarcoma, translational studies on paraffin-embedded tumors done independently by the Fred Hutchinson Cancer Center and Ohio State University have recently identified nearly uniform expression of NY-ESO-1 in virtually all (>90%) of cases examined [76,77]. As a member of the highly-immunogenic cancer testis family antigens, NY-ESO-1 has been successfully used as the target for immunotherapy in a number of solid tumors, including synovial sarcoma [78]. Of note, NY-ESO is not expressed in WD/DD liposarcoma [79] and expression in pleomorphic liposarcoma has not been reported. At the Fred Hutchinson Cancer Center, a phase I trial of autologous NY-ESO-1 specific T cells in unresectable or metastatic MRC liposarcoma is currently open and recruiting participants (NCT01477021). 

### 4.8. Other Potential Novel Systemic Therapies from Preclinical Studies

A number of other potential strategies for novel therapies in liposarcoma have not yet been tested, to our knowledge, in the setting of a clinical trial. Using large scale genomic analysis of multiple soft tissue sarcoma types, Barretina *et al*. identified YEATS4, a transcription factor involved in p53 regulation, as a potential target in WD/DD liposarcoma [80]. Knockdown of YEATS4 in liposarcoma cell lines resulted in better *in vitro* anti-proliferative effects compared to MDM2. The authors also identified a high frequency (18%) of mutations in PI3K in MRC liposarcoma. Patients with either kinase or helical domain mutations had worse disease specific survival, although the numbers of such patients were small. The PI3K/Akt pathways may also play an important role in the sarcomagenesis of WD liposarcoma. Gutierrez *et al*. used a zebrafish model to show that constitutive expression of Akt2 in mesenchymal progenitor cells resulted in development of adipocytic tumors that histologically resemble human WD liposarcoma [81]. In looking at patient samples, 27% of WD/DD liposarcoma cases examined demonstrated aberrant Akt activation. Small molecule inhibition of this pathway using BEZ235 demonstrated a dose dependent decrease in human liposarcoma cell viability and an increase in cell cycle arrest and apoptosis. These results are supported by findings recently reported by Smith *et al*., in which use of rapamycin, a PI3K/AKt/mTOR inhibitor, induced terminal differentiation and had anti-proliferative effects in a mouse xenograft model of DD liposarcoma [82]. Several other interesting candidate targets for novel systemic therapies, including c-jun, JNK and others, have been reported are comprehensively reviewed elsewhere [3,4].

## 5. Discussion/Conclusions

The reported results of recent clinical trials for novel systemic therapies in advanced liposarcoma are overall encouraging. With several novel therapies, disease stabilization is seen with relatively low toxicity. Trabectedin in MRC liposarcoma, flavopiridol in WD/DD liposarcoma, and sunitinib in subtype-unspecified liposarcoma appear to have fairly comparable or higher PFR than what has been reported for conventional cytotoxic chemotherapy. Objective responses are even seen in some patients, particularly with trabectedin in MRC liposarcoma. The caveat, of course, is that the current data is based on phase I and early phase II trials with relatively low numbers of patients (Table 2). More mature, phase II and III data in larger patient cohorts are clearly needed to make any definitive conclusions or alter current use of cytotoxic chemotherapy as first-line therapy. 

For the majority of novel therapies, treatment efficacy is heavily dependent on histologic subtype. A better understanding of characteristic genetic and molecular aberrations for each histologic subtype, has led to many of the novel therapies discussed, which directly target these aberrations. Clear disparity, however, exists in the level of development of novel systemic therapies among the three liposarcoma subtypes. In contrast to MRC liposarcoma, which already has known sensitivity to radiation therapy [11] and conventional cytotoxic chemotherapy [15,16] and now, trabectedin, novel therapies are poorly developed and have not been meaningfully evaluated in human studies for pleomorphic liposarcoma, the most aggressive histologic subtype. Barretina *et al*. reported that up to 17% of cases exhibit mutations in p53 [80]. More laboratory based studies are needed to better understand these mutations and how to best target them, as well as to identify other potential targets. For pleomorphic liposarcoma, this is of course challenging given its inherent genetic complexity and the overall rarity of this subtype. 

Although clinical trials in liposarcoma should ideally be based on histologic subtype, sufficient enrollment of patients within a limited period of time can be very challenging. With the exception of trabectedin and eribulin, all of the other novel systemic therapies have been evaluated in trials with at most twenty patients (Table 2). We strongly advocate referral to specialized centers to consolidate patient distribution and expedite enrollment. Specialized centers have the resources and laboratory support needed to appropriately analyze tumor response and validate potential biomarkers. This is particularly important in neoadjuvant trials, which allow for molecular proof-of-mechanism by analysis of the resected tumor in treated patients. Specialized centers are also best poised to study tumors from patients with progression of disease while receiving trial therapy, which is equally critical to understanding tumor resistance and escape mechanisms. Ultimately, for larger scale trials (e.g., phase III), multi-institutional and even intercontinental group collaborations between specialized centers are needed. 

In liposarcoma it is also important to define the pattern and extent of disease burden at the time of enrollment in a clinical trial. Even within the same histologic subtype, the broad category of “advanced disease” may include an isolated but unresectable tumor involving the root of the small bowel mesentery, intra-abdominal sarcomatosis, or distant visceral organ metastasis (e.g., lung) without locoregional recurrence; yet, each of these situations represents a unique disease biology with potentially different responses to therapy. Although it may not be practical to design a liposarcoma trial which enrolls only patients with a specific pattern or extent of disease, an attempt to meticulously document this may permit more meaningful subgroup analyses to determine which disease patterns may or may not respond to novel therapy. Recognition of lack of treatment efficacy for a specific disease pattern may also suggest exploration of alternative methods of drug delivery (e.g., local or regional therapy for the patient with a non-metastatic, but unresectable tumor).

Although all of the trials in this review focused on a single novel systemic therapy, combination approaches within a given histologic subtype should also be explored. Within each histologic subtype of liposarcoma, the multiple key genetic and molecular aberrations are often found together in the same tumor (e.g., MDM2 and CDK4 amplification in WD/DD liposarcoma). Therefore analogous to conventional cytotoxic chemotherapy, combinations of novel therapies may lead to synergistic effects that result in better disease stabilization and possibly, objective response. Combining molecular based therapy with immunotherapy may increase response rates in a durable fashion, leading to improved overall survival [83].

In conclusion, we feel that this is an exciting time for the development of biology-driven, novel systemic therapies for liposarcoma. The sheer abundance of clinical trials testing these biology-driven novel therapies truly exemplifies the “bench to bedside” translation of laboratory based findings to patient treatment, even for a relatively rare solid tumor. In recent years, several unique liposarcoma subtype-specific cell lines and experimental model systems have also been reported, which will certainly enhance preclinical testing of novel therapies [71,82,84,85,86,87]. With thoughtful design and conduct of clinical trials, we are hopeful that the near future will bring an emergence of several robust treatment options for the liposarcoma patient with advanced disease.
